# Relationship between effective and demographic population size in continuously distributed populations

**DOI:** 10.1111/eva.12636

**Published:** 2018-05-20

**Authors:** Jennifer C. Pierson, Tabitha A. Graves, Sam C. Banks, Katherine C. Kendall, David B. Lindenmayer

**Affiliations:** ^1^ Fenner School of Environment and Society The Australian National University Canberra ACT Australia; ^2^ ACT Parks and Conservation Service Environment and Planning and Sustainable Development Directorate Tharwa ACT Australia; ^3^ Northern Rocky Mountain Science Center United States Geological Survey West Glacier Montana

**Keywords:** effective population size, genetic indicator, genetic monitoring, LDNe, population trends

## Abstract

Genetic monitoring of wild populations can offer insights into demographic and genetic information simultaneously. However, widespread application of genetic monitoring is hindered by large uncertainty in the estimation and interpretation of target metrics such as contemporary effective population size, *N*_*e*_. We used four long‐term genetic and demographic studies (≥9 years) to evaluate the temporal stability of the relationship between *N*_*e*_ and demographic population size (*N*_*c*_). These case studies focused on mammals that are continuously distributed, yet dispersal‐limited within the spatial scale of the study. We estimated local, contemporary *N*_*e*_ with single‐sample methods (LDNE, Heterozygosity Excess, and Molecular Ancestry) and demographic abundance with either mark–recapture estimates or catch‐per‐unit effort indices. Estimates of *N*_*e*_ varied widely within each case study suggesting interpretation of estimates is challenging. We found inconsistent correlations and trends both among estimates of *N*_*e*_ and between *N*_*e*_ and *N*_*c*_ suggesting the value of *N*_*e*_ as an indicator of *N*_*c*_ is limited in some cases. In the two case studies with consistent trends between *N*_*e*_ and *N*_*c*_, *F*_IS_ was more stable over time and lower, suggesting *F*_IS_ may be a good indicator that the population was sampled at a spatial scale at which genetic structure is not biasing estimates of *N*_*e*_. These results suggest that more empirical work on the estimation of *N*_*e*_ in continuous populations is needed to understand the appropriate context to use LDNe as a useful metric in a monitoring programme to detect temporal trends in either *N*_*e*_ or *N*_*c*_.

## 1 INTRODUCTION

Genetic monitoring of populations has the potential to provide valuable information about both genetic and demographic population parameters (Schwartz, Luikart, & Waples, 2007). In some instances, demographic information can be gleaned directly from the genetic data such as using individual genotypes to conduct a capture–mark–recapture study to estimate population size (Kendall et al., 2009). In other instances, genetic metrics can be used as indicators of demographic parameters such as population size (Tallmon et al., 2010). However, the use of genetic metrics to track population trends is impeded by the challenges in interpreting common population genetic indicators targeted for monitoring (Pierson, Luikart, & Schwartz, 2015).

Effective population size (*N*_*e*_) is a metric of central interest in conservation biology, as it reflects information about ecological and evolutionary processes affecting populations (Luikart, Ryman, Tallmon, Schwartz, & Allendorf, 2010). A large and growing literature is dedicated to improving the accuracy of estimates of *N*_*e*_ (Do et al., 2014; Hare et al., 2011; Luikart et al., 2010; Neel et al., 2013; Tallmon, Waples, Gregovich, & Schwartz, 2012; Tallmon et al., 2010; Wang, 2009; Waples & Do, 2008; Waples, Luikart, Faulkner, & Tallmon, 2013) and the relationship between *N*_*e*_ and *N*_*c*_ (Palstra & Fraser, 2012; Waples, 2005; Waples et al., 2013). Yet, in most empirical applications, the true value of both *N*_*e*_ and *N*_*c*_ is unknown, and thus, the degree of uncertainty in the relationship between *N*_*e*_ and *N*_*c*_ is also unknown.

Despite all the challenges in estimating *N*_*e*_ in wild populations, the value of using genetic monitoring and thus identifying consistent relationships between contemporary local *N*_*e*_ and *N*_*c*_ has been clearly articulated (Luikart et al., 2010; Palstra & Fraser, 2012; Tallmon et al., 2010). The goal of monitoring is temporal, to track change over time, while assessment can be a snapshot of one moment in time (Schwartz et al., 2007). With genetic data now widely available for populations and the high cost of obtaining reliable estimates of *N*_*c*_ for wide‐ranging and difficult to count species, there is increased interest in the reliability of estimates of trends in *N*_*e*_ to provide indices of trends in *N*_*c*_ (Tallmon et al., 2012). Often, the target of monitoring is to determine whether abundance is increasing, decreasing or stable through time, with true abundance of less consequence. Consequently, the relationship between *N*_*e*_ and *N*_*c*_ within populations must be stable (or at least predictable) over time for *N*_*e*_ to be an informative indicator for *N*_*c*_ in a monitoring programme. Temporal fluctuations in *N*_*e*_ */N*_*c*_ ratio, potentially due to biased estimates, may hinder the use of *N*_*e*_ as an indicator of *N*_*c*_ (Tallmon et al., 2012).

Broadly, the concept of effective population size is defined as the size of an ideal population (e.g., constant population size, even sex ratio) that experiences genetic drift at the same rate as the observed population (Wright, 1931). In practice, the concept of effective population size varies at both temporal and spatial scales (Schwartz, Tallmon, & Luikart, 1998). Temporally, long‐term *N*_*e*_ approximately reflects the harmonic mean of effective population size over the last 4* *N*_*e*_ generations (Hare et al., 2011), whereas contemporary *N*_*e*_ reflects recent generations (Hare et al., 2011; Ovenden et al., 2007), and is the concept most relevant to genetic monitoring of population‐level processes.

Numerous methods exist to calculate *N*_*e*_ (Schwartz et al., 1998), including a range of approaches using one‐ or two‐sample genetic methods, such as sampling at multiple times to estimate *N*_*e*_ (Wang, 2005). In recent years, single‐sample genetic methods to estimate contemporary local *N*_*e*_. (Neel et al., 2013; Palstra & Fraser, 2012) have been developed including methods based on an excess of heterozygotes (Pudovkin, Zaykin, & Hedgecock, 1996), molecular coancestry (Nomura, 2008) and linkage disequilibrium (Hill, 1981; Waples & Do, 2008).

Practically, estimating *N*_*e*_ in natural populations is challenged by the fact that “real” populations often violate the rather restrictive assumptions of the idealized population scenarios around which *N*_*e*_ estimators have been developed, including overlapping generations (Waples, Antao, & Luikart, 2014), complex mating systems (Waples et al., 2013) and closed populations (Waples & England, 2011). Indeed, most natural populations share many of these attributes, often resulting in biased estimates of *N*_*e*_.

A particularly challenging situation is identifying the relationship between *N*_*e*_ and *N*_*c*_ in continuously distributed species (Neel et al., 2013), as genetic structure can bias estimates of *N*_*e*_ due to the Wahlund effect (Neel et al., 2013; Ryman, Allendorf, Jorde, Laikre, & Hossjer, 2014). Simulations suggest that including data from structured populations often result in severely biased underestimates of *N*_*e*_, for both the temporal method (Ryman et al., 2014) and the single‐sample linkage disequilibrium method (LDNe; Neel et al., 2013) and influences the *N*_*e*_/*N*_*c*_ ratio. Neel et al. (2013) suggest that *F*_IS_ can be used as an indicator of spatial structure that might bias *N*_*e*_. Specifically, they found that *F*_IS_ was negative when the sampling scale was smaller than the neighbourhood and positive when the sampling scale was larger than the neighbourhood. Indeed, Neel et al.'s (2013) simulations suggested that even a small positive *F*_IS_ value (~0.02) indicated significant underestimates of *N*_*e*_ due to the Wahlund effect. While simulation environments are excellent for forming hypotheses and testing theoretical predictions, empirical tests are needed to evaluate whether simulated results demonstrating *F*_IS_ can indicate genetic neighbourhood size consistently over time are supported in wild populations that experience multiple ecological and evolutionary pressures simultaneously. Additionally, stochasticity in sample size occurs in monitoring even when methods are consistent over time.

In this study, we empirically evaluate a range of monitoring scenarios in wild populations to gain greater understanding of empirical relationships between *N*_*e*_ and *N*_*c*_ to inform the appropriate use of *N*_*e*_ as an indicator of *N*_*c*_. We used empirical demographic and genetic monitoring data sets to investigate whether temporal patterns in *N*_*e*_ can function as a consistent indicator for temporal patterns in *N*_*c*_ and whether these patterns are sensitive to parameterization used for *N*_*e*_ estimation. Within the context of this aim, we also investigated spatial genetic structure within each study population as the presence of spatial genetic structure violates assumptions underpinning *N*_*e*_ estimation in wild populations (Neel et al., 2013).

We investigated the relationship between trends in estimates of *N*_*e*_ and trends in estimates of *N*_*c*_ using case studies of three mammals with sampling spanning nine to 22 years in duration (one – 10+ generations). Mammals are a common target of genetic monitoring given their elusive nature and ease of obtaining genetic samples (e.g., noninvasive hair snags and scat collection). We include empirical studies that include a range of demographic monitoring techniques that reflect realistic monitoring scenarios. All of these studies feature some limitations imposed by ecological and logistical reality, yet are representative of high‐quality data sets resulting from intensive survey effort over a long time period. These scenarios include robust mark–recapture estimates of population size and catch‐per‐unit effort which is an index of population size. Thus, the results of this study broadly inform the ability of *N*_*e*_ to be used as a genetic indicator of *N*_*c*_ in a genetic monitoring programme.

## 2 METHODS

We used three case studies from two continents where genetic and demographic monitoring of mammals had occurred for ≥9‐year time period: mountain brushtail possum (*Trichosurus cunninghami*), brown antechinus (*Antechinus stuartii*) and grizzly bear (*Ursus arctos*) at two different spatial scales and time spans. Demographic abundance is represented in a range of ways from a catch‐per‐unit effort (CPUE) index to capture–mark–recapture (CMR) estimates. For the purposes of this study, we refer to both indices and estimates as *N*_*c*._

### 2.1 Brown antechinus

The brown antechinus is a carnivorous marsupial found in coastal and montane ecosystems of south‐eastern Australia. This species is polyandrous and individuals typically live for 1 year and breed annually only once, although a very small proportion of females may survive to breed in the second year. Thus, they have discrete generations equal to a generation length of 1 year. Individuals weigh 16–44 g and females give birth to 6–7 young at the end of the annual breeding season in July/August. While the foraging home range is <1 ha, the social home range (most relevant to mate choice) of males is ~ 5 ha and for females is ~ 3 ha (Lazenby‐Cohen & Cockburn, 1991). The study population was part of a larger continuous population.

#### 2.1.1 Study population

The study area covered ~6,500 ha within Booderee National Park in south‐east Australia. This study had 129 permanent sampling sites each consisting of one 100‐m transect along which 10 Elliot aluminium box traps were deployed. From 2003 to 2012, 72–129 sites were sampled annually. Each site had a consistent effort of 30 trap nights (10 traps opened for three consecutive nights). Individuals were temporarily marked with a white paint pen, and an ear biopsy was taken from a subset of captures for DNA analysis. A demographic abundance index was estimated as catch‐per‐unit area by dividing the number of captures per session by the number of sites trapped per session.

DNA was extracted, and individuals were genotyped at 12 microsatellite loci: Aa1A, Aa2B, Aa2E, Aa2G, Aa2H, Aa4A, Aa4D, Aa4K, Aa7D, Aa7F, Aa7H and Aa7M. Primer sequences and PCR conditions for genotyping can be found in Banks et al. (2005), and forward primers for each locus were labelled with a M13 tag sequence for fluorescent labelling (Schuelke, 2000) prior to sequencing on an ABI3130 sequencer and genotype scoring on GeneMapper software. Testing for departures of genotype frequencies from Hardy–Weinberg expectations was conducted in Genepop (v. 4.2), and Aa2E, Aa4A and Aa7H were removed from the data set due to consistently significant departures from H‐W proportions.

### 2.2 Mountain brushtail possum

The mountain brushtail possum is a semi‐arboreal, omnivorous marsupial that inhabits wet sclerophyll forest in south‐eastern Australia and is a generalist herbivore. Individuals weigh 2.5–4.0 kg, exhibit a mixed mating system (polygamy with a proportion of monogamous individuals (Blyton, Banks, Peakall, & Lindenmayer, 2012)) and can live up to 12 years. Females typically produce one offspring per year (Banks, Knight, Dubach, & Lindenmayer, 2008), and a have generation length of 4 years (Blyton et al., 2012). Home range varies from an average of 2.6 ha for den tree use (Lindenmayer, Welsh, & Donnelly, 1997) to upwards of 30 ha for foraging range (Berry, Lindenmayer, Dennis, Driscoll, & Banks, 2016). The study population is part of a larger continuous population distributed across the Central Highlands of Victoria, Australia. The data set for this case study is described in full detail in Banks et al. (2015).

#### 2.2.1 Study population

The study area for the mountain brushtail possum covered 50 ha and was comprised of a 55‐trap grid. Between 1992 and 2013, 36 three‐night trapping sessions were completed where individual animals were uniquely marked. During the study, 263 individual animals were marked for a total of 1017 capture records. Abundance estimates were derived from capture numbers and recapture probability estimates from open population capture–mark–recapture estimates of survival, recruitment and recapture rate in MARK (White & Burnham, 1999) using the methods of Pradel (Pradel, 1996).

Genetic data for this population were available for all captured individuals genotyped at 16 autosomal microsatellite loci (Tv19, Tv27, Tv58, Tv64, TvM1, Tv.PnMs16, Tv5.64, MTcu3, MTcu9, MTcu11, MTcu27, MTcu29, MTcu30, MTcu31, MTcu34 and MTcu42) as described in Blyton, Shaw, and Banks (2014). The loci used were selected from a larger panel developed for the species after testing for departures of genotype frequencies from Hardy–Weinberg expectations, null alleles and linkage disequilibrium in the study population (Blyton et al., 2014).

### 2.3 Grizzly bears

Grizzly bears are large, iteroparous, polyandrous mammals that typically live up to 25 years in the wild. In this population, average age of first reproduction was 5.4 years, mean litter size was 2.27 (range 1–3) (Mace et al., 2012), and generation length of ~ 10 years is likely (Kamath et al., 2015). Adult bears in north‐western Montana range from 200 kg to 450 kg (Costello, Mace, & Roberts, 2016) with maximum male home range sizes exceeding 1,100 km^2^ (Mace and Waller 1997).

#### 2.3.1 Study population—Glacier National Park (GNP)

Sampling in the 410,000 ha area of GNP occurred each year 1998–2000, 2004 and 2009–2012. Barbed wire was placed on naturally occurring rub objects (most commonly trees) to sample bear hairs and genetic techniques were used to identify individuals (Kendall et al., 2009). A portion of the specific bear rubs varied between years, but the distribution of surveyed rubs was similar between years such that large portions of the GNP population had the opportunity to be detected each year. An index of demographic abundance was estimated as catch‐per‐unit effort by dividing the number of individuals detected by the summed number of days all rubs were available to accumulate. Therefore, grizzly bear catch‐per‐unit estimates are demographic indices of *N* that may be affected by interannual variation in detection that could influence the number of animals caught.

We used 7‐locus individual microsatellite genotypes that were consistent across sampling years for these analyses: G1A, G10J, G10M, G10P (Paetkau, Calvert, Stirling, & Strobeck, 1995), G10B, G1D (Paetkau & Strobeck, 1994) and G10H (Paetkau, Strobeck, & Shields, 1998). Genotyping details can be found in Kendall et al. (2009). Across all years, all loci in GNP met Hardy–Weinberg expectations.

#### 2.3.2 Study population—Northern Continental Divide Ecosystem (NCDE)

We genetically sampled grizzly bears across the full extent of the Northern Continental Divide Ecosystem population in north‐western Montana (3,141,000 ha) in 2004 and each year from 2009 to 2012. As in our GNP study, we placed barbed wire on naturally occurring rub trees and other objects to sample bear hairs and used microsatellite genotyping to identify individual bears (Kendall et al., 2009). An index of demographic population size was estimated as for the GNP study area; the number of individuals detected divided by the number of days on which rubs were available to accumulate hair.

After being designated threatened in 1975, the NCDE population was managed for recovery and the population has grown from an estimated 765 individuals in 2004 (Kendall et al., 2009) to approximately 946–1089 individuals in 2014 (Costello et al., 2016). Unlike the other case studies presented here, the NCDE grizzly bear population has six weakly separated subpopulations, although the genetic distance among them has declined and genetic diversity in more isolated subpopulations is increasing (Kendall et al., 2009; Mikle, Graves, Kovach, Kendall, & Macleod, 2016). Reflecting this, two of seven loci did not meet Hardy–Weinberg expectations and displayed an excess of homozygosity in some of the temporal samples (years).

### 2.4 Genetic parameter estimates

Effective population size was estimated using the one‐sample linkage disequilibrium method (Hill, 1981), the heterozygosity excess method (Pudovkin et al., 1996) and the molecular ancestry method (Nomura, 2008) in NeEstimator, (Do et al., 2014; Waples & Do, 2008). The linkage disequilibrium method assumes that the nonrandom associations between alleles among loci are due to genetic drift (Schwartz et al., 1998). The heterozygosity excess method assumes that in small populations there is variance in allele frequencies among male and females causing an excess of heterozygotes in the progeny (Pudovkin et al., 1996). The molecular ancestry method uses information on the relatedness of individuals in the sample to estimate *N*_*e*_ (Nomura, 2008). We varied two input parameters, the rare allele cut‐off and mating system to assess their influence on *N*_*e*_ estimates across time. We used three critical values to remove rare alleles (see Table S1 for allele frequencies per locus within each case study), 1/2S (S = smallest sample in data set), 1/2S (S = median sample size in data set) and 0.05, based on the optimal outcomes in previous simulation work (Waples & Do, 2008). While the brown antechinus is likely close to random mating, mountain brushtail possum and grizzly bears exhibit mixed mating systems (Blyton et al., 2012; Mikle et al., 2016). Therefore, we estimated *N*_*e*_ based on both random mating and monogamy assumptions. However, as these estimates are 100% correlated (exactly twofold for monogamy compared to random), we present the results from random mating only. Both the heterozygosity excess method and the molecular ancestry method produced results with high uncertainty (Table S2). Therefore, only the results from the linkage disequilibrium method are discussed further. We estimated inbreeding coefficients (*F*_IS_) for each trapping session in FSTAT.

### 2.5 Spatial genetic structure

We used spatial autocorrelation analyses based on multilocus genetic distances to determine the degree and pattern of spatial genetic structure within the study populations using GenAlEx 6.41 (Peakall & Smouse, 2006). We calculated the genetic autocorrelation coefficient (*r*) for approximately even‐sized distance classes and used bootstrapping (*n* = 999) to calculate 95% error bars around each estimate, assuming significance when the error bar did not cross zero. We removed six individuals from the mountain brushtail possum data set that did not have spatial information. For grizzly bears, we assessed autocorrelation with the same distance classes for both the GNP and NCDE case studies to compare the scale of spatial structure. Permutation tests (*n* = 999) were used to calculate a 95% confidence envelope. Significant isolation by distance was inferred when the estimate of *r* fell outside the confidence envelope around the null hypothesis of *r *=* *0. Permutation tests provide a robust estimate of significance when sample sizes are small because they use the entire data set (Peakall & Smouse, 2006).

### 2.6 Relationship between *N*_*e*_ and *N*_*c*_

We standardized all estimates of *N*_*c*_ and *N*_*e*_ to have a mean of zero and a standard deviation of one prior to further analyses to allow comparisons among data sets. We estimated Pearson's correlation coefficient (ρ) to quantify the relationship between short‐term fluctuations in *N*_*c*_ and *N*_*e*_. We tested the correlation between *N*_*e*_ and *N*_*c*_ of the previous generation to reflect that LDNe estimates the effective number of breeders in the previous reproductive cycle (i.e., parental generation; Waples, 2005). For antechinus, we used a generation length of 1 year. For mountain brushtail possums, we used a generation length of 4 years and reduced the data set to surveys a year apart where *N*_*e*_ estimates could be paired with an estimate of *N*_*c*_ approximately 4 years previously (*N* = 9). We also tested the correlation between *N*_*e*_ and *N*_*c*_ of the same year. Given the generation length of grizzly bears is ~10 years (Kamath et al., 2015), we did not have enough data to evaluate correlations between *N*_*e*_ and *N*_*c‐generation time*_.

To determine whether temporal trends in *N*_*e*_ and *N*_*c*_ were similar in each population, we tested for differences in the slopes of the predicted population trend from linear regressions between each abundance index and year with analysis of covariance (R v. 3.2.2). We first standardized each population estimate or index by subtracting the mean and dividing by the standard deviation to allow comparison of slopes (i.e., predicted population trends) across the population trend metrics. We used the *R*‐squared from individual models to evaluate the support for the existence of a population trend.

## 3 RESULTS

### 3.1 Are temporal patterns in *N*_*e*_ a consistent indicator for temporal patterns in *N*_*c*_, and are these patterns sensitive to parameterization of *N*_*e*_ estimation?

**Figure 1:**
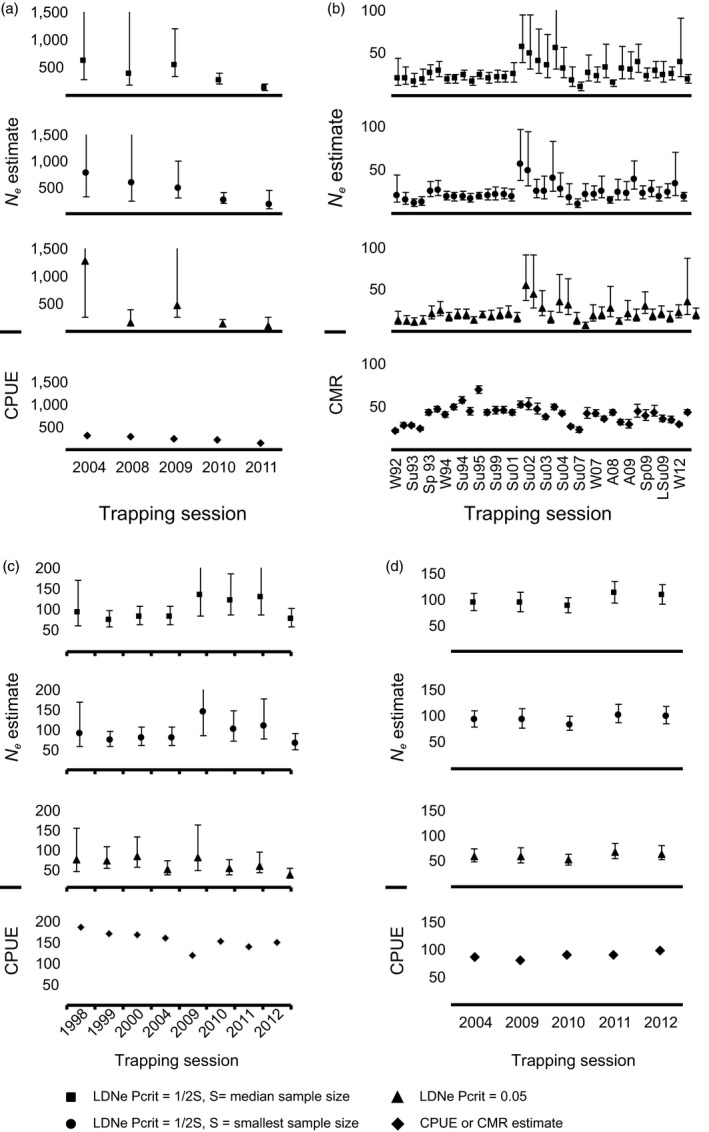
Estimates with 95% confidence intervals per method, including LDNe estimates with three different parameters used to remove rare alleles (square: Pcrit = 1/2S, S = median sample size; circle = 1/2S, S = smallest sample size; triangle: Pcrit = 0.05) diamond: CPUE or CMR estimate) and CPUE or CMR estimates for abundance (diamond); CPUE indices do not have errors associated with them. Full error estimates are included in Table 1 and Table S4. (a) Brown antechinus, (b) mountain brushtail possum, (c) Grizzly bears in Glacier National Park and (d) Grizzly bears in the Northern Continental Divide Ecosystem

The relationship between demographic population estimates and *N*_*e*_ varied depending on which estimate of *N*_*e*_ was used and the year of *N*_*c*_ (Figure 1, Table 2). We found large variation in the estimates of *N*_*e*_ within case studies depending on the critical value used to remove rare alleles from the data set (Figure 1, Table 1). For example, in 1 year, point estimates of *N*_*e*_ for brown antechinus varied from 615 to 1,271 across critical values. Correlation among estimates of *N*_*e*_ based on the range of critical values used for rare allele cut‐off also varied within case studies (Table S3). The brown antechinus case study had consistently high correlations between *N*_*e*_ and *N*_*c‐generation time*_ (CPUE; ρ = .84:.96) and between *N*_*e*_ and *N*_*c*_ (ρ = .61:.96) (Table 2). The mountain brushtail possum case study had low correlations between *N*_*e*_ and *N*_*c*_ (ρ = .29:.39), *N*_*c‐1*_ (ρ = .18:.30) and *N*_*c‐generation time*_ (ρ = −.02:.39), with the lowest correlations between *N*_*e*_ matched to the 4‐year generation length lag time. The correlation between *N*_*e*_ and *N*_*c*_ varied most widely in the GNP grizzly bear case study across critical values (ρ = −.72:.37) (Table 2).

**Table 1:** (a) Brown antechinus. (b) Mountain brushtail possum. (c) Grizzly bears in Glacier National Park. (d) Grizzly bears in the Northern Continental Divide Ecosystem.^a^ Sample sizes per trapping session and estimates of effective population size (*N_e_*) calculated in NeEstimator using the LDNe method (random mating). Estimates are based on critical values for rare allele cutoff of 1/2S (S = smallest sample size), 1/2S (S = median sample size), 0.05, Confidence intervals are parametric 95% confidence intervals

(a) Trapping session	Sample size	Harmonic mean sample size	LDNe (0.004)	Lower 95% CI	Higher 95% CI	LDNe (0.007)	Lower 95% CI	Higher 95% CI	LDNe (0.05)	Lower 95% CI	Higher 95% CI
2004	141	136.2	615.1	297.2	15,076	767	330.6	Inf	1,271	255.7	Inf
2008	106	104.3	378.2	196.4	2,152	578.4	244	Inf	149.4	85.9	378.5
2009	221	217.5	551.5	344.2	1,209	485.9	308.4	1,004	466.3	244.5	1,968
2010	212	209.9	272.4	201	400.3	267.7	196.5	396.4	139.5	99.2	210.1
2011	75	74.9	121.3	82.5	207.7	174.2	102.9	445.2	100.4	57.9	247.4

(b) Trapping session	Sample size	Harmonic mean sample size	LDNe (0.018)	Lower 95% CI	Higher 95% CI	LDNe (0.03)	Lower 95% CI	Higher 95% CI	LDNe (0.05)	Lower 95% CI	Higher 95% CI
W92	16	15.9	20.8	12.4	44.6	20.8	12.4	44.6	13.4	8.4	23.7
Sp92	21	20.8	20.5	13.7	33.9	16.1	11.1	24.9	12.4	8.6	18.5
Su93	20	19.9	16.9	11.7	26.1	11.5	8.1	16.8	11.5	8.1	16.8
A93	20	20	19	13.6	31.8	13.1	9.4	19.1	13.1	9.4	19.1
Sp 93	32	31.9	26.1	19.4	36.8	26.1	19.4	36.8	21.5	15.6	30.9
A94	36	35.9	29.4	22.3	40.3	27.4	20.4	38.4	25.6	19	36.2
W94	33	32.9	19.4	15.1	25.5	19.4	15.1	25.5	17.3	13	23.6
Sp94	37	36.9	19.8	15.7	25.5	18.9	14.9	24.3	19.9	15.3	26.3
Su94	41	40.9	24.1	19.2	30.9	19.6	15.4	25.2	20.1	15.5	26.7
A95	28	27.9	16.9	12.8	22.8	16.8	12.8	22.7	13.4	10.1	18.2
Su95	49	48.9	24	19.6	29.8	19.8	16.2	24.3	19.8	16	24.8
Su98	32	31.7	20.1	15.1	27.7	20.1	15.1	27.7	18.4	13.6	25.4
Su99	30	30	21.6	15.9	31	21.6	15.9	31	20	14.6	28.8
Su00	35	34.9	21.6	16.5	28.9	21.5	16.4	29.1	22.3	16.7	30.8
Su01	24	23.9	24.8	17	39.8	19.2	13.8	28.4	16.1	11.6	23.6
A02	40	39.7	57.6	39.6	95.5	57.3	38.7	97.7	55.2	37.7	92.4
Su02	30	29.9	49.1	31.4	95.4	49.1	31.4	95.4	45.9	28.9	92.1
A03	26	25.9	40.7	26.1	78.4	25.4	17.7	40	28.3	18.7	49.6
Su03	23	22.5	35.5	22.1	72.3	25.1	16.6	43.3	15.5	10.5	24.2
A04	27	26.2	55.3	31.9	149.3	40.4	25.1	83	35.6	22.5	69.1
Su04	26	25.6	32.3	21.2	57.3	28.5	19.3	47.4	32.1	20.1	63.4
Su05	16	16	17.7	11	33.9	17.7	11	33.9	13.3	8.5	22.6
Su07	16	15.7	10.2	6.6	16.2	10.2	6.6	16.2	7.2	4.3	11
A07	23	22.3	27.2	17.7	48.7	21.3	14.5	34.5	19.4	12.9	32.2
W07	28	27.4	23.2	16.6	34.7	21.6	15.6	31.7	20.2	14.6	29.5
Su08	24	23.5	32.6	21	61	25.9	17.6	42.8	28.6	18.2	54
A08	31	30.7	15	11.7	19.5	15	11.7	19.5	12.7	9.8	16.6
W08	23	22.4	31.6	20.4	58.4	23.8	16.1	39.5	21.8	14.5	36.8
A09	23	22.7	24.6	16.8	40.1	19.9	13.8	31	16.6	11.7	24.9
W09	23	22.9	29.8	19.9	51.8	23.2	16	37	17.9	12.5	27.4
Sp09	33	32.4	39	27.5	61.1	39	27.5	61.1	30.6	21.4	47.6
Su09	31	30	23.3	17.4	32.6	23.2	17.3	32.5	19.4	14.3	27.2
LSu09	34	33.3	28.6	21.1	40.8	26.7	19.7	38.1	21.8	16.2	30.6
Sp10	31	30.4	24.7	18.4	34.6	24.7	18.4	34.6	22.5	16.7	31.6
W12	22	21.6	39.1	23.3	91.4	34	20.9	71	35.6	20.6	88.2
W13	33	32.8	18.8	14.5	24.8	18.8	14.5	24.8	20.7	15.5	28.6

(c) Trapping session	Sample size	Harmonic mean sample size	LDNe (0.004)	Lower 95% CI	Higher 95% CI	LDNe (0.007)	Lower 95% CI	Higher 95% CI	LDNe (0.05)	Lower 95% CI	Higher 95% CI
1998	69	69	90.8	58.5	170.2	90.8	58.5	170.2	75.7	46.3	154.8
1999	140	140	73.2	57.5	95.6	73.2	57.5	95.6	74	53	108.7
2000	134	134	80	62	106.5	80	62	106.5	83.1	56.6	132.3
2004	127	127	79.7	61.3	107.4	79.7	61.3	107.4	52	37.9	73.8
2009	84	84	133.9	83.4	277.6	143.8	86.1	333.9	80.3	48.7	163.7
2010	119	119	119.8	84.7	186.2	100.3	72.9	147.6	53.2	38.6	76.2
2011	102	102	127.6	86.2	217.9	110.7	77.3	176.2	60.6	41.8	94.4
2012	106	106	74.4	56.7	101.8	65.9	50.4	89.6	38.6	28.6	53.3

(d) Trapping session	Sample size	Harmonic mean sample size	LDNe (0.001)	Lower 95% CI	Higher 95% CI	LDNe (0.002)	Lower 95% CI	Higher 95% CI	LDNe (0.05)	Lower 95% CI	Higher 95% CI
2004	275	275	93.3	78.6	111.7	91.8	77.1	110.1	59	47.2	74
2009	248	248	93.1	76.8	114.1	93.1	76.8	114.1	58.9	46	75.7
2010	330	330	87.8	74.5	103.9	83	70.6	97.8	51.2	41.6	62.9
2011	318	318	111.6	93.5	134.3	101.5	85.6	121.1	67.1	54.2	83.3
2012	344	344	108.3	91.5	129	99.5	84.3	118.1	63.4	51.1	78.8

a Note that Ne estimates are biased low in the NCDE due to substructure, as evidenced by similar *N*_*e*_ than in GNP (nested within NCDE).

**Table 2:** Pearson's correlation coefficients estimated between demographic estimates of population size and effective population size. Demographic estimates include catch‐per‐unit effort (CPUE) and capture–mark–recapture (CMR). Effective population size was estimated using the LDNe method with input parameters that included a range of values of rare allele cut‐offs (in parentheses) based on a random mating system. Correlations were tested between the current year estimates of *N*_*e*_, and generation length for brown antechinus (1 year) and mountain brushtail possum (4 years). The 10‐year generation length of grizzly Bears precludes the ability to compare using generation length

	Brown antechinus		Mountain brushtail possum		GNP grizzly bear	Northern Continental Divide Ecosystem grizzly bear
	CPUE	CPUE 1‐year lag	CMR estimate	CMR 4‐year lag	CPUE	CPUE
LDNe (1/2S median)	.851	.839	.290	−.017	−.722	.548
LDNe (1/2S smallest)	.957	.956	.298	.056	−.713	.297
LDNe (0.05)	.662	.916	.385	.034	.159	.260

**Figure 2:**
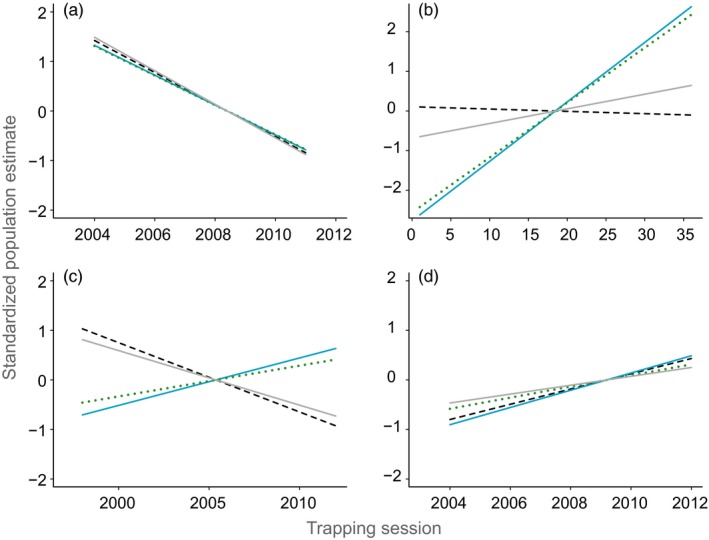
Predicted population trajectories estimated from linear models based on standardized estimates of *N*_*e*_ (based on a range of values to remove rare alleles from data set) and *N*_*c*_ (based on either a capture–mark–recapture estimate for mountain brushtail possum or a catch‐per‐unit effort index for brown antechinus and grizzly bears). The *x*‐axis is trapping session, and the *y*‐axis is standardized estimates of *N*_*e*_ and *N*_*c*_. (a) Brown antechinus, (b) mountain brushtail possum, (c) GNP grizzly bears and (d) Northern Continental Divide Ecosystem (NCDE) grizzly bears. Blue solid line: LDNe Pcrit = 1/2S, S = median sample size; green dotted line: LDNe Pcrit = 1/2S, S = smallest sample size; grey solid line: LDNe Pcrit = 0.05; black dashed line: CMR or CPUE

In three of the four case studies, slopes of *N*_*e*_ regressed by year were not significantly different from slopes of *N*_*c*_ regressed by year (Figure 2; Table 3): brown antechinus (*F* = 0.03, *p* = .99), mountain brushtail possum (*F* = 0.51, *p* = .77) and NCDE grizzly bears (*F* = .05, *p* = .99). However, the direction of trends varied across metrics in the mountain brushtail possum case study (Figure 2, Table 3). In the GNP grizzly bear study, the slopes of estimates of trend were significantly different: GNP grizzly bears (*F* = 3.55, *p* = .01) and the direction of trend varied among estimates (Figure 2, Table 3).

**Table 3:** Results from linear regression analysis estimating trend in population abundance from beginning to end of each study. Individual models were run for each abundance metric

a. Brown antechinus					
	Estimate	*SE*	*t* value	*p* > *t*	*R*‐squared
CPUE	−.324	0.104	−3.124	**.052**	.687
LDNe (0.004)	−.301	0.124	−2.428	.094	.550
LDNe (0.007)	−.299	0.126	−2.364	.099	.534
LDNe (0.05)	−.338	0.087	−3.893	**.030**	.780

b. Mountain brushtail possum					
	Estimate	*SE*	*t* value	*p* > *t*	*R*‐squared
Abundance	−.006	0.011	−0.540	.593	−.021
LDNe (0.018)	.150	0.122	1.231	.227	.015
LDNe (0.03)	.139	0.109	1.268	.213	.017
LDNe (0.05)	.037	0.030	1.229	.227	.014

c. Grizzly bears in Glacier National Park					
	Estimate	*SE*	*t* value	*p* > t	*R*‐squared
CPUE	−.140	0.041	−3.411	**.014**	.603
LDNe (0.004)	.096	0.058	1.639	.152	.194
LDNe (0.007)	.062	0.066	0.948	.380	−.015
LDNe (0.05)	−.110	0.054	−2.037	.088	.310

d. Grizzly bears in the Northern Continental Divide Ecosystem					
	Estimate	*SE*	*t* value	*p* > *t*	*R*‐squared
CPUE	.154	0.163	0.945	.414	−.027
LDNe (0.001)	.174	0.156	1.116	.346	.058
LDNe (0.002)	.112	0.174	0.645	.565	−.171
LDNe (0.05)	.090	0.178	0.503	.649	−.230

Bold *p*‐values indicate significant directional trends.

### 3.2 Spatial genetic structure within monitoring units

We found significant spatial genetic structure as measured by spatial autocorrelation within all four case studies (Figure S1). All four case studies showed a pattern expected of isolation by distance where individuals geographically closer together are more related than individuals further apart. We were not able to evaluate the effect of structure on the uncertainty in estimates of *N*_*e*_ as the true value of *N*_*e*_ and *N*_*c*_ remains unknown.

**Figure 3:**
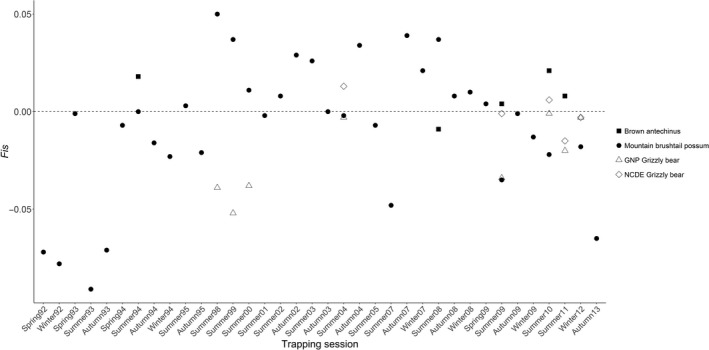
Temporal patterns in estimates of the inbreeding coefficient (*F*_IS_) in each study system

Estimates of *F*_IS_, calculated as (1—observed heterozygosity/expected heterozygosity), varied from year to year within each case study although the spatial extent of sampling in each study was constant (Figure 3). The brown antechinus and NCDE grizzly bear case studies both had *F*_IS_ within a small range of ≤.02 (range −.01:.02; −.015:.013 respectively), despite the presence of spatial structure within the NCDE study area (Kendall et al., 2009). Mountain brushtail possums and the GNP grizzly bear case studies had *F*_IS_ values ranging from −.09 to .05 and −.05 to 0, respectively (Figure 3).

## 4 DISCUSSION

Effective population size has the potential to be a highly informative metric for monitoring trends in population abundance, as it provides information on both ecological and evolutionary processes. However, *N*_*e*_ as a concept is fundamentally linked to assumptions of ideal populations (e.g., random mating, constant population size, equal sex ratio), and estimates are complicated by several factors such as the presences of overlapping generations (Waples, 2005) and spatial genetic structure (Gilbert & Whitlock, 2015; Neel et al., 2013; Waples & England, 2011). Yet most wild populations are often complex and display many of these characteristics. Hence, *N*_*e*_ is notoriously difficult to accurately estimate and interpret in wild populations. We found that estimates of contemporary *N*_*e*_ varied widely within each case study depending on the critical value used to remove rare alleles, and the temporal trends among estimates of *N*_*e*_ and *N*_*c*_ were only consistent in two of the four case studies.

The two case studies with a consistent relationship between trends in *N*_*e*_ and *N*_*c*_, brown antechinus and NCDE grizzly bears, both had a relatively stable, narrow range of estimates of *F*_IS._ While simulations (Neel et al., 2013) suggest that *F*_IS_ is a good indicator of the spatial scale at which to estimate local *N*_*e*_ (or Wright's genetic neighbourhood), our empirical results support that high temporal variation, that is, low temporal stability in *F*_IS_, may be an indicator that the study is not at the appropriate spatial scale to estimate *N*_*e*_. We suggest that if the objective is to use genetic estimates of *N*_*e*_ to indicate trends in abundance for a particular area, temporal stability in *F*_IS_ may be an indicator of the consistency of association between *N*_*e*_ and *N*_*c*_ and therefore the suitability of *N*_*e*_ as an indicator of trends in abundance. *F*_IS_ is known to be affected by a variety of factors including mating system, *N*_*e*_ itself, gene flow and spatial population structure, and thus, high temporal variability in *F*_IS_ may indicate a disconnect between trends in *N*_*e*_ and *N*_*c*_ due to other biological processes.

### 4.1 Temporal patterns in *N*_*e*_ as an indicator of temporal patterns in *N*_*c*_

We found a strong relationship between trends in *N*_*c*_ and *N*_*e*_ in brown antechinus and NCDE grizzly bears, regardless of the critical value used for rare allele removal. However, in mountain brushtail possums and GNP grizzly bears, the direction and strength of trends varied between *N*_*e*_ and *N*_*c*._ This suggests that trends in *N*_*e*_ can perform quite well as an indicator of trends in *N*_*c*_ but not in all cases. The inconsistency between the two nested grizzly bear case studies suggests that spatial scale of sampling may at times play a role in the relationship between trends in *N*_*e*_ and *N*_*c*_. Surprisingly, trends between *N*_*e*_ and *N*_*c*_ were consistent in the larger NCDE study area where spatial structure is known to exist (Kendall et al., 2009; Mikle et al., 2016) and were not consistent in the smaller study area with less substantial structure. The GNP study area covered many bear home ranges, but covered only approximately half of two closely related “subpopulations” within the larger NCDE metapopulation. Similar to the larger NCDE study area, the brown antechinus study area was relatively large compared to their home range and showed consistent trends between *N*_*e*_ and *N*_*c*._ The mountain brushtail possum study area was relatively small compared to their home range and showed inconsistent trends between *N*_*e*_ and *N*_*c*_ however, the effect was not significant. The lack of detection of an effect could be in part due to high natural variability in *N* and the limited directional trend observed. These case studies suggest that large sampling areas, relative to the home range of species, may reflect local *N*_*e*_ in continuous populations even when substructure is present, but may not be reliable when only part of a subpopulation is sampled. Therefore, caution should be used when trying to use trends in *N*_*e*_ to indicate trends in *N*_*c*_ without knowledge of the prior relationship between the two metrics within the study area.

Specific estimates of *N*_*e*_ and *N*_*c*_ varied markedly within each case study, and estimates for both CPUE and *N*_*e*_ were imprecise. The uncertainty in *N*_*e*_ estimates is difficult to remedy as the results of this study indicate that both the value used to remove rare alleles from the data set and the selection of mating system have a large influence on the estimate of *N*_*e*_. The challenge with this is that relatively few species have strictly monogamous or random mating systems (Reynolds, 1996). If the aim was simply to monitor trends over time, the bias caused by mating system is consistent and therefore not of concern. However, the inconsistent bias caused by the effects of rare alleles on estimates of *N*_*e*_ leads to challenges in interpreting temporal trends.

### 4.2 *F*_IS_ as an indicator of spatial structure

We would expect *F*_IS_ to be relatively stable through time if *F*_IS_ a good indicator of the appropriate scale of the sampling genetic neighbourhood when the spatial extent is consistent across time (Neel et al., 2013). Specifically, even slightly positive estimates of *F*_IS_ may indicate the Wahlund effect in simulations, which occurs when genetically diverged individuals are included in the same sample (Neel et al., 2013). This can occur when the sampling window is greater than the breeding window. However, these simulations were carried out under relatively narrow conditions.

We found that *F*_IS_ was relatively stable in the two case studies that had stable relationships between trends in *N*_*e*_ and *N*_*c*_, (brown antechinus and the NCDE grizzly bear case studies). It is possible that the consistent relationship found in these case studies is related to sampling at a spatial scale that is appropriate to estimate *N*_*e*_. The grizzly bear case studies provide an example of two spatial scales of sampling, and interestingly, the larger scale with known substructure displayed similar trends in *N*_*e*_ and *N*_*c*_. Further examples of cases studies that provide similar comparisons among spatial scales of sampling are needed to explore these patterns in more depth.

### 4.3 Implications for genetic monitoring

Many species are continuously distributed across large spatial areas that span many land management and political boundaries. However, genetic monitoring programmes are often designed and implemented at spatial scales that reflect the goal of a local programme, such as management or project boundary, regardless of genetic structure. Additionally, the spatial scale of random mating or genetic neighbourhood size is often unknown prior to designing the programme and can vary over time or in response to landscape disturbances (Banks et al., 2015). Such was the case with the studies herein which spanned a range of spatial scales. Each monitoring programme was initially established with a goal reflected in the spatial scale of sampling. For example, work on brown antechinus is part of a monitoring study conducted within the boundaries of Booderee National Park; the goal of the monitoring study was to elucidate patterns associated with habitat variables across a landscape (Lindenmayer et al., 2016). Thus, the sampling design and scale reflect this goal.

Previous simulation and empirical work suggest that in some cases the relationship between *N*_*e*_ and *N*_*c*_ may be consistent enough for *N*_*e*_ to be useful for monitoring. Tallmon et al. (2012) conducted a simulation study to determine whether *N*_*e*_ could perform as a useful index of *N*_*c*_ and found that *N*_*e*_ can detect trends in abundance when population sizes are small and when *N*_*e*_/*N*_*c*_ ratios are low. However, they caution that temporal fluctuations in the *N*_*e*_/*N*_*c*_ ratio may occur due to factors such as variance in reproductive success.

In wild populations, there have been mixed results in evaluating the relationship between *N*_*e*_ and *N*_*c*_. Previous work on grizzly bears near Yellowstone National Park (Kamath et al., 2015) found congruency between *N*_*e*_ and *N*_*c*_. That study sampled bears across the full extent of the target population, which is not known to exhibit substructure. Studies on brown trout (*Salmo trutta*) (Charlier, Laikre, & Ryman, 2012) and amphibians (Nunziata, Scott, & Lance, 2015) found that the methods used to calculate *N*_*e*_ determined whether there was a correlation with demographic abundance. In contrast, empirical estimates of *N*_*e*_ did not reflect changes or trends in *N*_*c*_ in brook trout, but rather reflected population‐specific individual reproductive contributions (Whiteley et al., 2015). Similarly, a study on a hatchery‐supplemented river‐dwelling fish, the Rio Grande silvery minnow *(Hybognathus amarus),* found a lack of correlation among temporal estimates of *N*_*c*_, and *N*_*e*_ (Osborne, Carson, & Turner, 2012). A meta‐analyses of empirical estimates of *N*_*e*_/*N*_*c*_ ratios revealed that a log‐linear relationship between *N*_*e*_ and *N*_*c*_ was a better fit than a linear relationship, suggesting that *N*_*e*_ is informative about *N*_*c*_ in very small populations (Palstra & Fraser, 2012). Such discrepancies in the relationship between *N*_*e*_ and *N*_*c*_ are not surprising given that both ecological and evolutionary pressures may affect both *N*_*c*_ and *N*_*e*_ simultaneously, resulting in divergent patterns in each metric over time. Additionally, these studies reflect a range of approaches to estimate demographic abundance which may introduce more variation in the relationship between effective and demographic population size due to the uncertainty in both individual estimates.

Effective population size is affected by factors other than demographic population size (Charlesworth, 2009). Thus, management actions that change demographic abundance may not change *N*_*e*_. This was the case in Rio Grande silvery minnow which experienced large fluctuations in demographic abundance due to hatchery stocking, yet these fluctuations were not reflected in *N*_*e*_. The value of *N*_*e*_ as an indicator of *N*_*c*_ may be highest when the factors that differentially affect *N*_*e*_ and *N*_*c*_ are minimized.

An extensive literature is directed at improving the accuracy and precision of estimates of both demographic and effective population size (Palstra & Fraser, 2012). Our results support the lack of precision in estimates of *N*_*e*_ as well as suggesting that true values are difficult to estimate due to the large influence of rare alleles. We suggest that in some instances, such as when the outcome of interest is an estimate of the trend in population size, that perhaps highly precise estimates of either *N*_*e*_ or *N*_*c*_ are unnecessary for some objectives. However, our study found that trends in *N*_*e*_ do not always indicate similar trends in *N*_*c*._

## 5 CONCLUSIONS

The stable temporal relationship among estimates of and trends in *N*_*e*_ and *N*_*c*_ found in some of our case studies suggests that *N*_*e*_ may sometimes be a good metric for monitoring when the goal was to detect changes over time as opposed to tracking “true” population size. However, caution must be used as, even within a species, *N*_*e*_ and *N*_*c*_ do not consistently show similar patterns and trends over time. This could be due to variation in the spatial scale of sampling in these populations, violations of the assumptions behind estimates of *N*_*e*_ (e.g., overlapping generations), changes in the variance of reproductive success that could result from changes in population sizes where density dependence is a factor, or a variety of other reasons such as uncertainty in estimates of *N*_*c*_. Thus, more evaluation of long‐term monitoring data sets such as these is needed for further insight into the appropriate use of effective population size as an indicator of demographic population size.

This study takes a simple correlative approach to evaluating the usefulness of trends in *N*_*e*_ as an indicator of trends in demographic population size in long‐term empirical data sets. While there are limitations to correlative evidence, simple tests can provide useful guidance on the empirical relationship between metrics over time (Osborne et al., 2012; Pierson et al., 2016) and may be useful in applying to real monitoring programmes (Hoban et al., 2014). We note that the implications of this study are limited to mammal populations that have a relatively small *N*_*e*_ and have not been subject to any management actions that would substantially alter reproductive variance, such as those directed towards population supplementation (e.g., hatchery fish) or removal (e.g., harvest). More empirical evaluations of different scenarios (e.g., species, growth trajectories, threats) are needed to continue to inform our empirical understanding the relationship between *N*_*e*_ and *N*_*c*_ over time in natural populations.

## CONFLICT OF INTEREST

We do not have any conflict of interests.

## DATA AVAILABILITY

Grizzly bear data is available at ScienceBase.gov (Mikle et al. 2016); mountain brushtail possum and brown antechinus data available at the Long Term Ecological Research Network's data portal (ltern.org.au).
