# Supplementary figures and images for: Relationship between effective and demographic population size in continuously distributed populations
Source: Evol Appl. 2018 May 20;11(7):1162–75. doi: 10.1111/eva.12636 (PMC6050178; doi:10.1111/eva.12636)

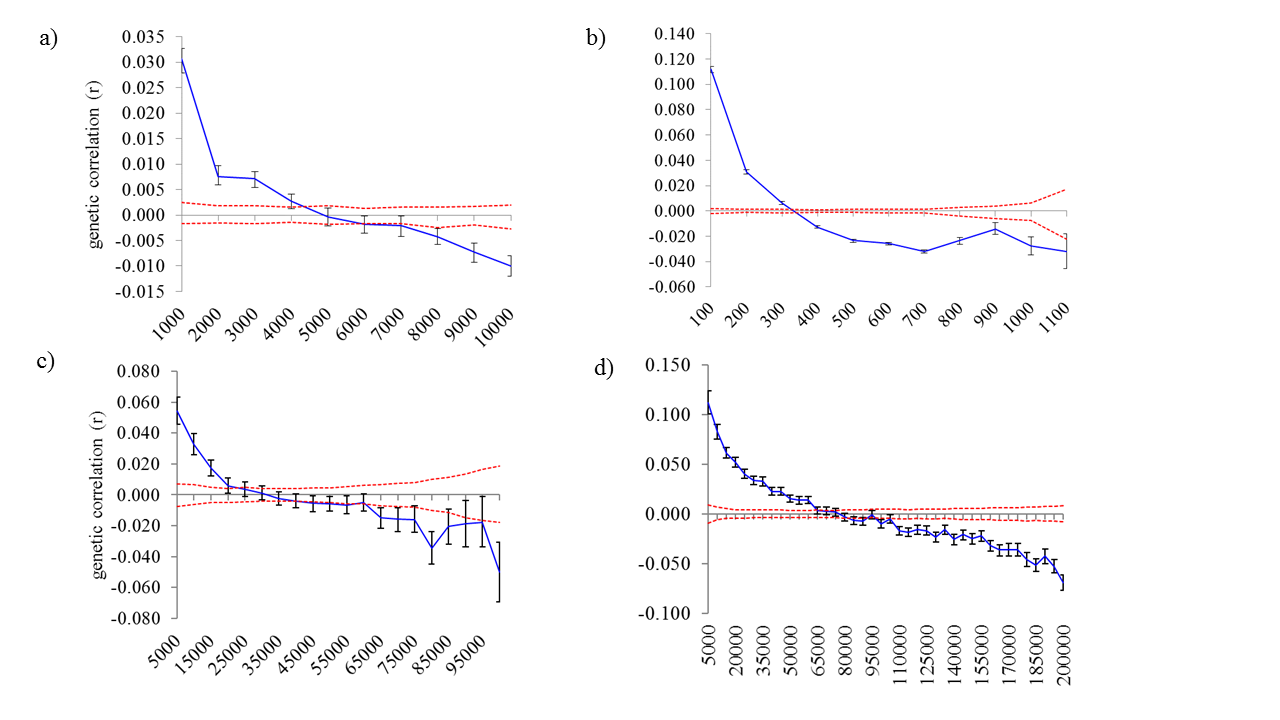

Supplement: Supplementary file 1 [file EVA-11-1162-s001.tif]
