# Supplementary material for: Relationship between effective and demographic population size in continuously distributed populations
Source: Evol Appl. 2018 May 20;11(7):1162–75. doi: 10.1111/eva.12636 (PMC6050178; doi:10.1111/eva.12636)
Supplement: Supplementary file 2 [file EVA-11-1162-s002.docx]

Table S1. Allele frequencies per locus in each case study pooled across temporal samples.

1. Brown antechinus

| Locus | A1 | A2 | A3 | A4 | A5 | A6 | A7 | A8 | A9 | A10 | A11 | A12 | A13 | A14 | A15 | A16 |
| --- | --- | --- | --- | --- | --- | --- | --- | --- | --- | --- | --- | --- | --- | --- | --- | --- |
| Aa1A | 0.495 | 0.001 | 0.074 | 0.091 | 0.011 | 0.026 | 0.007 | 0.04 | 0.044 | 0.039 | 0.073 | 0.088 | 0.007 | 0.003 | ­ | ­ |
| Aa2B | 0.009 | 0.021 | 0.592 | 0.197 | 0.074 | 0.019 | 0.046 | 0.043 | ­ | ­ | ­ | ­ | ­ | ­ | ­ | ­ |
| Aa2G | 0.974 | 0.014 | 0.013 | ­ | ­ | ­ | ­ | ­ | ­ | ­ | ­ | ­ | ­ | ­ | ­ | ­ |
| Aa2H | 0.002 | 0.042 | 0.013 | 0.027 | 0.054 | 0.099 | 0.001 | 0.161 | 0.17 | 0.132 | 0.03 | 0.002 | 0.038 | 0.075 | 0.146 | 0.007 |
| Aa4D | 0.053 | 0.001 | 0.08 | 0.115 | 0.197 | 0.094 | 0.021 | 0.094 | 0.18 | 0.143 | 0.023 | ­ | ­ | ­ | ­ | ­ |
| Aa4K | 0.007 | 0.01 | 0.969 | 0.014 | ­ | ­ | ­ | ­ | ­ | ­ | ­ | ­ | ­ | ­ | ­ | ­ |
| Aa7D | 0.002 | 0.051 | 0.183 | 0.022 | 0.411 | 0.191 | 0.088 | 0.045 | 0.001 | 0.007 | ­ | ­ | ­ | ­ | ­ | ­ |
| Aa7F | 0.808 | 0.001 | 0.047 | 0.004 | 0.003 | 0.001 | 0.104 | 0.034 | ­ | ­ | ­ | ­ | ­ | ­ | ­ | ­ |
| Aa7M | 0.186 | 0.148 | 0.168 | 0.122 | 0.179 | 0.17 | 0.028 | ­ | ­ | ­ | ­ | ­ | ­ | ­ | ­ | ­ |

1. Mountain brushtail possum

| Locus | A1 | A2 | A3 | A4 | A5 | A6 | A7 | A8 | A9 | A10 | A11 |
| --- | --- | --- | --- | --- | --- | --- | --- | --- | --- | --- | --- |
| MTcu031 | 0.003 | 0.98 | 0.017 | ­ | ­ | ­ | ­ | ­ | ­ | ­ | ­ |
| MTcu011 | 0.011 | 0.001 | 0.839 | 0.149 | ­ | ­ | ­ | ­ | ­ | ­ | ­ |
| MTcu042 | 0.164 | 0.252 | 0.483 | 0.088 | 0.002 | 0.012 | ­ | ­ | ­ | ­ | ­ |
| MTcu027 | 0.101 | 0.188 | 0.606 | 0.106 | ­ | ­ | ­ | ­ | ­ | ­ | ­ |
| Tv5.64 | 0.056 | 0.358 | 0.586 | ­ | ­ | ­ | ­ | ­ | ­ | ­ | ­ |
| MTcu003 | 0.016 | 0.6 | 0.375 | 0.009 | ­ | ­ | ­ | ­ | ­ | ­ | ­ |
| MTcu029 | 0.003 | 0.294 | 0.149 | 0.489 | 0.064 | ­ | ­ | ­ | ­ | ­ | ­ |
| MTcu034 | 0.018 | 0.155 | 0.78 | 0.047 | ­ | ­ | ­ | ­ | ­ | ­ | ­ |
| MTcu030 | 0.192 | 0.519 | 0.033 | 0.256 | ­ | ­ | ­ | ­ | ­ | ­ | ­ |
| MTcu009 | 0.479 | 0.079 | 0.037 | 0.116 | 0.042 | 0.046 | 0.133 | 0.067 | 0.001 | ­ | ­ |
| Tv_PnMs16 | 0.001 | 0.136 | 0.002 | 0.505 | 0.015 | 0.122 | 0.072 | 0.01 | 0.084 | 0.004 | 0.047 |
| Tv19 | 0.324 | 0.091 | 0.457 | 0.128 | ­ | ­ | ­ | ­ | ­ | ­ | ­ |
| Tv27 | 0.531 | 0.396 | 0.022 | 0.035 | 0.015 | ­ | ­ | ­ | ­ | ­ | ­ |
| Tv58 | 0.006 | 0.234 | 0.347 | 0.194 | 0.137 | 0.083 | ­ | ­ | ­ | ­ | ­ |
| Tv64 | 0.037 | 0.092 | 0.07 | 0.185 | 0.279 | 0.058 | ­ | ­ | ­ | ­ | ­ |
| TvM1 | 0.319 | 0.264 | 0.392 | 0.024 | ­ | ­ | ­ | ­ | ­ | ­ | ­ |

1. GNP Grizzly bears

| Locus | A1 | A2 | A3 | A4 | A5 | A6 | A7 | A8 | A9 | A10 | A11 | A12 |
| --- | --- | --- | --- | --- | --- | --- | --- | --- | --- | --- | --- | --- |
| G10J | 0.124 | 0.277 | 0.367 | 0.065 | 0.167 | ­ | ­ | ­ | ­ | ­ | ­ | ­ |
| G1A | 0.076 | 0.205 | 0.044 | 0.084 | 0.469 | 0.001 | 0.12 | ­ | ­ | ­ | ­ | ­ |
| G10B | 0.011 | 0.076 | 0.131 | 0.056 | 0.04 | 0.302 | 0.03 | 0.323 | 0.031 | ­ | ­ | ­ |
| G1D | 0.001 | 0.094 | 0.026 | 0.008 | 0.275 | 0.161 | 0.011 | 0.039 | 0.062 | 0.293 | 0.023 | 0.006 |
| G10H | 0.016 | 0.186 | 0.04 | 0.461 | 0.035 | 0.086 | 0.053 | 0.093 | 0.03 | ­ | ­ | ­ |
| G10M | 0.003 | 0.21 | 0.033 | 0.356 | 0.364 | 0.017 | 0.004 | 0.014 | ­ | ­ | ­ | ­ |
| G10P | 0.025 | 0.354 | 0.373 | 0.062 | 0.075 | 0.08 | 0.03 | ­ | ­ | ­ | ­ | ­ |

1. NCDE Grizzly bears

| Locus | A1 | A2 | A3 | A4 | A5 | A6 | A7 | A8 | A9 | A10 | A11 | A12 |
| --- | --- | --- | --- | --- | --- | --- | --- | --- | --- | --- | --- | --- |
| G10J | 0.118 | 0.324 | 0.292 | 0.115 | 0.15 | 0 | ­ | ­ | ­ | ­ | ­ | ­ |
| G1A | 0.056 | 0.152 | 0.079 | 0.134 | 0.455 | 0.001 | 0.123 | ­ | ­ | ­ | ­ | ­ |
| G10B | 0.042 | 0.037 | 0.16 | 0.04 | 0.068 | 0.349 | 0.041 | 0.25 | 0.001 | 0.015 | ­ | ­ |
| G1D | 0.001 | 0.122 | 0.034 | 0.005 | 0.245 | 0.158 | 0.009 | 0.033 | 0.046 | 0.331 | 0.014 | 0.003 |
| G10H | 0.027 | 0.108 | 0.058 | 0.566 | 0.038 | 0.038 | 0.025 | 0.08 | 0.06 | 0 | 0 | ­ |
| G10M | 0.008 | 0.186 | 0.024 | 0.374 | 0.327 | 0.073 | 0.002 | 0.005 | ­ | ­ | ­ | ­ |
| G10P | 0.013 | 0.327 | 0.244 | 0.037 | 0.138 | 0.159 | 0.083 | ­ | ­ | ­ | ­ | ­ |

Table S2. Estimates of effective population size (*N_e_*) calculated in NeEstimator using the Heterozygosity Excess (HE) method, and Molecular Ancestry (Mol Coa) methods. Estimates are based on critical values for rare allele cutoff of 1/2S (S = smallest sample size), 1/2S (S = median sample size), 0.05, Confidence intervals are parametric 95% confidence intervals.

2a. Brown antechinus

| Session | HE (0.004) | Lower 95%CI | Higher 95%CI | HE (0.007) | Lower 95%CI | Higher 95%CI | HE (0.05) | Lower 95%CI | Higher 95%CI | Mol Coa | Lower 95%CI | Higher 95%CI |
| --- | --- | --- | --- | --- | --- | --- | --- | --- | --- | --- | --- | --- |
| 2004 | Inf | Inf | Inf | Inf | Inf | Inf | Inf | 26.2 | Inf | Inf | Inf | Inf |
| 2008 | 65.8 | 16.6 | Inf | 65.8 | 16.8 | Inf | 31.8 | 10 | Inf | 942.6 | 0.9 | 4732 |
| 2009 | 700.7 | 32.8 | Inf | 544.9 | 30.6 | Inf | Inf | 27.3 | Inf | Inf | Inf | Inf |
| 2010 | Inf | Inf | Inf | Inf | Inf | Inf | Inf | Inf | Inf | Inf | Inf | Inf |
| 2011 | Inf | 19.9 | Inf | Inf | 18 | Inf | Inf | 12.5 | Inf | Inf | Inf | Inf |

2b. Mountain brushtail possum

| Session | HE (0.018) | Lower 95%CI | Higher 95%CI | HE (0.03) | Lower 95%CI | Higher 95%CI | HE (0.05) | Lower 95%CI | Higher 95%CI | Mol Coa | Lower 95%CI | Higher 95%CI |
| --- | --- | --- | --- | --- | --- | --- | --- | --- | --- | --- | --- | --- |
| Winter92 | 7.1 | 4.5 | 17.7 | 7.1 | 4.5 | 17.7 | 6.4 | 4.2 | 15.3 | 12.7 | 1.5 | 35.3 |
| Spring92 | 8.9 | 5.3 | 31.7 | 8.5 | 5.1 | 29 | 7.7 | 4.7 | 25.1 | 12.9 | 2.7 | 31 |
| Summer93 | 7.4 | 4.8 | 17.2 | 7.5 | 4.5 | 24.1 | 7.5 | 4.5 | 24.1 | 11.2 | 2.3 | 27 |
| Autumn93 | 8.9 | 5.3 | 30.2 | 8.3 | 5 | 26.5 | 8.3 | 5 | 26.5 | 10.8 | 2.6 | 24.8 |
| Spring93 | 42.3 | 10.9 | Inf | 42.3 | 10.9 | Inf | 423.2 | 11.3 | Inf | 13 | 4.5 | 25.9 |
| Autumn94 | 33.6 | 10.8 | Inf | 40.7 | 10.4 | Inf | 47.8 | 10.3 | Inf | 8.7 | 4.1 | 15 |
| Winter94 | 27 | 9.8 | Inf | 27 | 9.8 | Inf | 55.9 | 9.9 | Inf | 10.1 | 3.5 | 20.2 |
| Spring94 | 32.9 | 11.7 | Inf | 89.4 | 12 | Inf | 274.6 | 12 | Inf | 8.2 | 4.4 | 13.1 |
| Summer94 | 47.9 | 14.4 | Inf | 37.6 | 12.3 | Inf | 130.2 | 13.3 | Inf | 19.2 | 2.3 | 53.5 |
| Autumn95 | 16.5 | 8.1 | Inf | 16.5 | 8.1 | Inf | 18.7 | 7.7 | Inf | 7.1 | 3.6 | 11.7 |
| Summer95 | 43.5 | 13.6 | Inf | 40.9 | 12.9 | Inf | 36.7 | 11.8 | Inf | 21.8 | 1.6 | 67.9 |
| Summer98 | Inf | 20.5 | Inf | Inf | 20.5 | Inf | Inf | 18.2 | Inf | 11.4 | 4.6 | 21.2 |
| Summer99 | Inf | 23.2 | Inf | Inf | 23.2 | Inf | Inf | 26.9 | Inf | 12.9 | 2.7 | 31 |
| Summer00 | 285.1 | 12.9 | Inf | 282.3 | 11.9 | Inf | Inf | 12.5 | Inf | 73.8 | 0.1 | 370.7 |
| Summer01 | 53.2 | 9.7 | Inf | 53.2 | 9.7 | Inf | 352.9 | 9.9 | Inf | Inf | Inf | Inf |
| Autumn02 | Inf | 31.1 | Inf | Inf | 37.3 | Inf | Inf | 37.3 | Inf | 12.6 | 3.8 | 26.6 |
| Summer02 | Inf | 14.6 | Inf | Inf | 14.6 | Inf | Inf | 15.2 | Inf | 32.7 | 0.8 | 120.5 |
| Autumn03 | Inf | 13.1 | Inf | Inf | 12.4 | Inf | Inf | 12.4 | Inf | 182.5 | 0.2 | 915.9 |
| Summer03 | Inf | 18.1 | Inf | Inf | 17 | Inf | Inf | 17.1 | Inf | 7.4 | 1.8 | 16.9 |
| Autumn04 | Inf | 27.9 | Inf | Inf | 26.8 | Inf | Inf | 24.7 | Inf | 15.6 | 1.1 | 48.5 |
| Summer04 | Inf | 11 | Inf | Inf | 11 | Inf | Inf | 10.9 | Inf | Inf | Inf | Inf |
| Summer05 | 168.2 | 7.8 | Inf | 168.2 | 7.8 | Inf | 107.6 | 7.4 | Inf | 9 | 3.8 | 16.5 |
| Summer07 | 18.5 | 5.9 | Inf | 18.5 | 5.9 | Inf | 17.1 | 4.5 | Inf | 96.4 | 0.1 | 484.1 |
| Autumn07 | Inf | 24.2 | Inf | Inf | 23.2 | Inf | Inf | 18.9 | Inf | 20.8 | 1.5 | 64.8 |
| Winter07 | Inf | 17.3 | Inf | Inf | 17.3 | Inf | Inf | 19.3 | Inf | 8.5 | 4.1 | 14.6 |
| Summer08 | Inf | 24.5 | Inf | Inf | 24.5 | Inf | Inf | 28.5 | Inf | 14.5 | 1.1 | 45.3 |
| Autumn08 | Inf | 15.5 | Inf | Inf | 15.5 | Inf | Inf | 14.2 | Inf | 9.2 | 3 | 18.9 |
| Winter08 | Inf | 13.8 | Inf | Inf | 12.7 | Inf | Inf | 15.5 | Inf | 17.8 | 2.1 | 49.7 |
| Summer09 | 35.1 | 7.9 | Inf | 30.5 | 7.3 | Inf | 29.9 | 7 | Inf | 171.9 | 0.2 | 862.7 |
| Autumn09 | 40 | 8.7 | Inf | 38.9 | 8.4 | Inf | 32.6 | 7.5 | Inf | 18.3 | 1.3 | 57 |
| Winter09 | Inf | 13.2 | Inf | Inf | 13.2 | Inf | Inf | 12.9 | Inf | Inf | Inf | Inf |
| Spring09 | Inf | 14.1 | Inf | Inf | 14.1 | Inf | Inf | 13.3 | Inf | Inf | Inf | Inf |
| Summer09 | 35.9 | 9.5 | Inf | 36.4 | 9.4 | Inf | 45.4 | 9.2 | Inf | 10.8 | 1.3 | 30 |
| LSummer09 | 35.1 | 7.9 | Inf | 30.5 | 7.3 | Inf | 29.9 | 7 | Inf | 171.9 | 0.2 | 862.7 |
| Summer10 | 26 | 7.8 | Inf | 26 | 7.8 | Inf | 23.3 | 5.9 | Inf | 20.8 | 0 | 104.5 |
| Winter12 | 39.9 | 7.7 | Inf | 37.5 | 7.5 | Inf | 17.2 | 6.6 | Inf | 67.7 | 0.1 | 340.1 |
| Winter13 | 17.7 | 6.6 | Inf | 17.7 | 6.6 | Inf | 11.5 | 6.2 | 172.2 | 30.2 | 0 | 151.7 |

2c. Grizzly bears in Glacier National Park.

| Session | HE (0.004) | Lower 95%CI | Higher 95%CI | HE (0.007) | Lower 95%CI | Higher 95%CI | HE (0.05) | Lower 95%CI | Higher 95%CI | Mol Coa | Lower 95%CI | Higher 95%CI |
| --- | --- | --- | --- | --- | --- | --- | --- | --- | --- | --- | --- | --- |
| 1998 | 18.8 | 9.8 | 422.7 | 18.8 | 9.8 | 422.7 | 16.6 | 8.1 | Inf | Inf | Inf | Inf |
| 1999 | 17 | 10 | 63.7 | 17 | 10 | 63.7 | 15.2 | 7.9 | 1460.8 | 83.1 | 0.1 | 417 |
| 2000 | 35.2 | 13.9 | Inf | 35.2 | 13.9 | Inf | 20.6 | 10 | Inf | Inf | Inf | Inf |
| 2004 | 434.1 | 22.6 | Inf | 434.1 | 22.6 | Inf | 92.4 | 15.4 | Inf | 29.8 | 3.6 | 83 |
| 2009 | 172.2 | 12.2 | Inf | 152.9 | 11.7 | Inf | 27.2 | 7.9 | Inf | Inf | Inf | Inf |
| 2010 | Inf | 35.2 | Inf | Inf | 34.4 | Inf | Inf | 19.1 | Inf | 23.9 | 1.8 | 74.5 |
| 2011 | 40.6 | 14.9 | Inf | 40.6 | 14.9 | Inf | 24.3 | 9.9 | Inf | 21.8 | 3.6 | 55.9 |
| 2012 | Inf | 26.7 | Inf | Inf | 24.8 | Inf | 151.6 | 12.9 | Inf | 32 | 0 | 160.9 |

2d. . Grizzly bears in the Northern Continental Divide Ecosystem

| Session | HE (0.001) | Lower 95%CI | Higher 95%CI | HE (0.002) | Lower 95%CI | Higher 95%CI | HE 0.05 | Lower 95%CI | Higher 95%CI | Mol Coa | Lower 95%CI | Higher 95%CI |
| --- | --- | --- | --- | --- | --- | --- | --- | --- | --- | --- | --- | --- |
| 2004 | Inf | 97.6 | Inf | Inf | 95.5 | Inf | Inf | 50.4 | Inf | Inf | Inf | Inf |
| 2009 | Inf | 46.7 | Inf | Inf | 46.7 | Inf | 134.2 | 22.4 | Inf | Inf | Inf | Inf |
| 2010 | Inf | 1838 | Inf | Inf | 1838 | Inf | Inf | 56.1 | Inf | Inf | Inf | Inf |
| 2011 | 191.5 | 24 | Inf | 191.5 | 24 | Inf | 61.3 | 16.5 | Inf | Inf | Inf | Inf |
| 2012 | Inf | 55.9 | Inf | Inf | 54.5 | Inf | 155 | 25.1 | Inf | Inf | Inf | Inf |

Table S3. Pearson’s correlation coefficients estimated among estimates of *N_e_* based on a range of critical values used to remove rare alleles from the dataset (in parentheses). Estimates are based on the LDNe method implemented in Neestimator and a random mating system was applied.

1. Brown antechinus

|  | LDNe (0.004) | LDNe (0.007) | LDNe (0.05) |
| --- | --- | --- | --- |
| LDNe (0.004) | 1.000 | 0.890 | 0.804 |
| LDNe (0.007) |  | 1.000 | 0.796 |
| LDNe (0.05) |  |  | 1.000 |

1. Mountain brushtail possum

|  | LDNe (0.018) | LDNe (0.03) | LDNe (0.05) |
| --- | --- | --- | --- |
| LDNe (0.018) | 1.000 | 0.926 | 0.893 |
| LDNe (0.03) |  | 1.000 | 0.951 |
| LDNe (0.05) |  |  | 1.000 |

1. Grizzly Bears in Glacier National Park

|  | LDNe (0.004) | LDNe (0.007) | LDNe (0.05) |
| --- | --- | --- | --- |
| LDNe (0.004) | 1.000 | 0.924 | 0.139 |
| LDNe (0.007) |  | 1.000 | 0.379 |
| LDNe (0.05) |  |  | 1.000 |

1. Grizzly Bears in the Northern Continental Divide Ecosystem

|  | LDNe (0.001) | LDNe (0.002) | LDNe (0.05) |
| --- | --- | --- | --- |
| LDNe (0.001) | 1.000 | 0.938 | 0.931 |
| LDNe (0.002) |  | 1.000 | 0.990 |
| LDNe (0.05) |  |  | 1.000 |

Table S4. Estimates of population size (*N*) of Mountain Brushtail Possums calculated using the Pradel model in program MARK. Estimates were obtained by dividing the number of individuals by probability of capture (separate for each sex); 95% CI calculated based on 95% CI of probability of capture.

| Session | CMR estimated abundance | Lower 95% CI | Upper 95% CI |
| --- | --- | --- | --- |
| Winter92 | 22.7 | 21.29 | 24.31 |
| Spring92 | 29.49 | 27.59 | 31.67 |
| Summer93 | 28.91 | 27.04 | 31.07 |
| Autumn93 | 25.56 | 23.89 | 27.5 |
| Spring 93 | 44.14 | 41.25 | 47.48 |
| Autumn94 | 47.63 | 44.39 | 51.41 |
| Winter94 | 41.06 | 38.2 | 44.4 |
| Spring94 | 50 | 46.74 | 53.78 |
| Summer94 | 58.63 | 54.87 | 62.95 |
| Autumn95 | 45.02 | 41.3 | 49.47 |
| Summer95 | 70.3 | 65.83 | 75.44 |
| Summer98 | 43.66 | 40.83 | 46.92 |
| Summer99 | 46.42 | 42.52 | 51.12 |
| Summer00 | 47.22 | 44.16 | 50.74 |
| Summer01 | 43.6 | 40.41 | 47.36 |
| Autumn02 | 52.94 | 49.3 | 57.17 |
| Summer02 | 53.48 | 47.67 | 60.91 |
| Autumn03 | 48.17 | 42.8 | 55.11 |
| Summer03 | 38.61 | 36.06 | 41.55 |
| Autumn04 | 50.51 | 47.25 | 54.26 |
| Summer04 | 43.46 | 40.71 | 46.62 |
| Summer05 | 27.93 | 25.95 | 30.23 |
| Summer07 | 23.39 | 20.39 | 27.43 |
| Autumn07 | 42.91 | 37.37 | 50.42 |
| Winter07 | 43.22 | 39.94 | 47.09 |
| Summer08 | 36.66 | 34.16 | 39.56 |
| Autumn08 | 43.61 | 40.67 | 47.02 |
| Winter08 | 32.37 | 29.04 | 36.57 |
| Autumn09 | 29.8 | 25.61 | 35.68 |
| Winter09 | 45.09 | 38.79 | 53.92 |
| Spring09 | 39.97 | 34.49 | 47.57 |
| Summer09 | 44.72 | 39.34 | 51.85 |
| LSummer09 | 36.67 | 33.17 | 41.01 |
| Summer10 | 35.25 | 31.8 | 39.56 |
| Winter12 | 30.41 | 28.55 | 32.53 |
| Winter13 | 44.34 | 41.72 | 47.38 |

Figure S1. Correlograms showing spatial structure present in each case study. The y-axis is genetic correlation (r) within each distance class on the x-axis (m). a) Brown antechinus, b) Mountain brushtail possum, c) GNP Grizzly bears, and d) NCDE Grizzly bears.
